# Analysis of Suicide After Cancer Diagnosis by US County-Level Income and Rural vs Urban Designation, 2000-2016

**DOI:** 10.1001/jamanetworkopen.2021.29913

**Published:** 2021-10-19

**Authors:** Ryan Suk, Young-Rock Hong, Rachel M. Wasserman, J. Michael Swint, N. Belinda Azenui, Kalyani B. Sonawane, Alexander C. Tsai, Ashish A. Deshmukh

**Affiliations:** 1 Center for Health Systems Research, Policy and Practice, Department of Management, Policy and Community Health, University of Texas Health Science Center at Houston School of Public Health; 2 Center for Health Promotion and Preventive Research, Department of Management, Policy and Community Health, University of Texas Health Science Center at Houston School of Public Health; 3 Department of Health Services Research, Management and Policy, College of Public Health and Health Professions, University of Florida, Gainesville; 4 UF Health Cancer Center, Gainesville, Florida; 5 Center for Healthcare Delivery Science, Nemours Children’s Health System, Orlando, Florida; 6 Center for Clinical Research and Evidence-Based Medicine, University of Texas Health Science Center at Houston McGovern School of Medicine; 7 Department of Economics, Denison University, Granville, Ohio; 8 Center for Health Services Research, Department of Management, Policy and Community Health, University of Texas Health Science Center at Houston School of Public Health; 9 Center for Healthcare Data, Department of Management, Policy and Community Health, University of Texas Health Science Center at Houston School of Public Health; 10 Center for Global Health and Mongan Institute, Massachusetts General Hospital, Boston; 11 Harvard Medical School, Boston, Massachusetts

## Abstract

**Importance:**

Studies suggest the risk of suicide among people with cancer diagnosis is higher compared with the general population. However, little is known about how suicide risk among people diagnosed with cancer might vary according to area-level income and rurality.

**Objective:**

To examine whether the risks and patterns of suicide mortality among people with a cancer diagnosis differ by US county-level median income and rural or urban status.

**Design, Setting, and Participants:**

A retrospective, population-based cohort study following up individuals who were diagnosed with cancer between January 1, 2000, and December 31, 2016, was conducted. The Surveillance, Epidemiology, and End Results Program 18 registries (SEER 18) database was used to obtain data on persons diagnosed with a first primary malignant tumor. Comparisons with the general US population were based on mortality data collected by the National Center for Health Statistics. Analyses were conducted from February 22 to October 14, 2020.

**Exposures:**

County-level median household income and urban or rural status.

**Main Outcomes and Measures:**

Standardized mortality ratios (SMRs) of suicide deaths and annual percentage changes (APCs) of SMRs.

**Results:**

The SEER 18 database included 5 362 782 persons with cancer diagnoses living in 635 counties. Most study participants were men (51.2%), White (72.2%), and older than 65 years (49.7%). Among them, 6357 persons died of suicide (SMR, 1.41; 95% CI, 1.38-1.44). People with cancer living in the lowest-income counties had a significantly higher risk (SMR, 1.94; 95% CI, 1.76-2.13) than those in the highest-income counties (SMR, 1.30; 95% CI, 1.26-1.34). Those living in rural counties also had significantly higher SMR than those in urban counties (SMR, 1.81; 95% CI, 1.70-1.92 vs SMR, 1.35; 95% CI, 1.32-1.39). For all county groups, the SMRs were the highest within the first year following cancer diagnosis. However, among people living in the lowest-income counties, the risk remained significantly high even after 10 or more years following cancer diagnosis (SMR, 1.83; 95% CI, 1.31-2.48). The comparative risk of suicide mortality within 1 year following cancer diagnosis significantly decreased over the years but then plateaued in the highest-income (2005-2015: APC, 2.03%; 95% CI, −0.97% to 5.13%), lowest-income (2010-2015: APC, 4.80%; 95% CI, −19.97% to 37.24%), and rural (2004-2015: APC, 1.83; 95% CI, −1.98% to 5.79%) counties.

**Conclusions and Relevance:**

This cohort study showed disparities in suicide risks and their patterns among people diagnosed with cancer by county-level income and rural or urban status. The findings suggest that additional research and effort to provide psychological services addressing these disparities among people with cancer may be beneficial.

## Key Points

**Question:**

Do US counties with differing levels of income and urbanicity vary in rates of suicide among people after cancer diagnosis?

**Findings:**

In this cohort study that included 5 362 782 people with a cancer diagnosis, there was a significantly higher suicide risk in those living in the lowest-income counties and in rural settings. The risk was highest in the first year after diagnosis and remained higher in those living in the lowest-income counties 10 or more years after diagnosis.

**Meaning:**

This study’s findings suggest that efforts to provide increased preventive mental health services for individuals with cancer, especially for those living in low-income and rural areas, are needed.

## Teaser

This cohort study examines the risk of suicide among US individuals with a cancer diagnosis by income and in rural and urban settings.

## Introduction

Despite recent improvements in rates of cancer survival, deaths from suicide among people with cancer continue to be higher than the general population in the US.^1,2,3,4^ Both individual-level sociodemographic (age, sex, economic status, and marital status) and clinical (depression, cancer site, and time since diagnosis) risk factors are associated with suicide among people with cancer.^2,3,5,6^ In particular, depression, one of the most common psychiatric complications among people with cancer, has been reported to increase the risk of mortality by nearly 40%.^7,8,9^ Although the determinants of depressive disorders among people diagnosed with cancer are likely multifactorial, some risk factors for suicide may be more prevalent among people with cancer: functional impairment, poorer social and family well-being, and lower socioeconomic status.^8,10^ Data also suggest financial insolvency contributes to early mortality among people with cancer. Ramsey et al^11^ reported that filing bankruptcy was associated with a 79% increase in early mortality among people with cancer. These findings imply that social and economic status may be important factors in understanding the higher rates of suicide among people with cancer.

Socioeconomic risk factors are often geographically clustered and can be associated with both intermediate (eg, depression and functional impairment) and terminal (ie, mortality) health outcomes.^12,13^ However, there is limited research elucidating how neighborhood and other contextual characteristics are associated with suicide among people with cancer. A better understanding of variation in suicide deaths by contextual social vulnerability could potentially identify modifiable risk factors amenable to policy or programmatic intervention. Therefore, we estimated suicide death rates after cancer diagnosis in the US, stratified by the county-level characteristics (median household income and rurality). We focused primarily on median household income and rural or urban status because previous studies assessing mental health care access at the ecological level have reported that area-level income and rurality are associated with a lack of mental health care clinicians.^14,15^ We hypothesized that suicide rates among people with cancer would be elevated in counties with lower median household income and in rural counties.

## Methods

### Data Sources

We conducted a retrospective population-based cohort study using the Surveillance, Epidemiology, and End Results Program 18 registries (SEER 18) database.^16^ SEER 18 provides population-based information on people with cancer collected from 18 registries in the US. For comparisons with the general US population, mortality data from the US National Center for Health Statistics were collected through SEER 18. County-level income and metropolitan status available in the SEER 18 database are derived from the American Community Survey (2000s). Data are deidentified and publicly available under a data use agreement with the US National Cancer Institute; thus, this study was deemed exempt from institutional review board approval by the University of Texas Health Science Center. Analyses were conducted from February 22 to October 14, 2020. This study followed the Strengthening the Reporting of Observational Studies in Epidemiology (STROBE) reporting guideline for cohort studies.

### Study Population and Variables of Interest

We included people diagnosed with a first primary malignant tumor between January 1, 2000, and December 31, 2016. They were followed up from cancer diagnosis until death due to suicide and self-inflicted injury, death due to other causes, date last known to be alive, or the end of follow-up (December 31, 2016), whichever came first. People whose cancer diagnoses were derived solely from death certificates or autopsy were excluded. Individuals were also excluded if their ages at the times of diagnosis were unknown.

Our primary outcome of interest was death due to suicide and self-inflicted injury identified using cause-of-death information. County-level income was defined by median household income in the county, and we then classified the counties into quartiles based on national percentiles across counties. We did not restrict the quartile estimation to the 18 registries because we wanted the quartiles to be based on the nationwide distribution of county income level, which would be more appropriate for identifying affluent counties. County-level rural and urban status was defined using the Rural-Urban Continuum codes from 2003.^17^ Nonmetropolitan counties (continuum codes 4-9) were classified as rural and metropolitan counties (codes 1-3) were classified as urban. In addition, the latency period between cancer diagnosis and death (0-11, 12-59, 60-119 and ≥120 months) and calendar year of cancer diagnosis were examined. Individual-level demographic variables were also included in stratified analyses: age (<40, 40-64, and ≥65 years), sex (men and women), and race and ethnicity (Hispanic, non-Hispanic Asian/Pacific Islander [hereafter, Asian/Pacific Islander], non-Hispanic Black [hereafter, Black], non-Hispanic White [hereafter, White], and non-Hispanic Other [American Indian/Alaska Native or Unknown; hereafter, Other]). This study used cancer registry data, and the race code in the registry data was extracted from the patients’ medical records (collected by health care facilities and practitioners). Race and ethnicity were included in our analysis as they may be reflective of social determinants of health and thus associated with health outcomes.

We explored patterns of suicide mortality by several additional county-level socioeconomic characteristics. These characteristics included educational level attainment (percentage of residents without a high-school diploma, with cutoffs specified at the national quartiles), unemployment rate (by quartile), and percentage of Black residents (with cutoffs specified at the national average [12.5%], 30%, and 50%).

### Statistical Analysis

We calculated standardized mortality ratios (SMRs) as the number of observed cases divided by the number of expected cases. Observed cases represent the number of suicide deaths within our study population (persons with a cancer diagnosis). Expected cases represent the number of suicide deaths in the general population with a similar demographic distribution within the same period. For estimating the number of expected cases, stratum-specific suicide mortality rates for the general reference population were derived, and the person-years of relevant strata in the cancer group were calculated. The 95% CIs for SMRs were obtained based on a Poisson regression model and considered statistically significant when the range did not overlap with 1.0. Excess absolute risks, which represent the absolute number of additional suicide deaths, were calculated as the excess number of suicide deaths (ie, the number of observed suicide deaths minus the number of expected suicide deaths) among people with cancer diagnosis per 10 000 person-years at risk.

First, we described the demographic characteristics of the study population, stratified by county-level attribute (income quartile and rural or urban status). Then we estimated comparative suicide risk, expressed as the SMR, stratified by county-level attributes. We also stratified by race and ethnicity, age, and sex within each county group to compare the risk of suicide across the county attributes and individual demographic factors. To examine whether the suicide risk varies by time since cancer diagnosis across the county-level attributes, we estimated SMRs by latency period across county-level income and rural/urban status. To understand the temporal trends of suicide risk by county-level attributes, we first calculated SMRs for suicide during the first year after the cancer diagnosis for each calendar year from 2000 to 2016. We then estimated annual percentage changes (APCs), average annual percentage changes (AAPCs), and *P* values using joinpoint regression. We restricted the temporal analysis to the suicide risks within the first year following the cancer diagnosis to minimize lag-time bias from the differences in follow-up years by calendar year of cancer diagnosis (eg, overall SMR of cancer diagnosis in 2015 will represent only slightly more than 1 year of the latency period and thus will be overestimated compared with the overall SMR of 2000 because the first 1-year suicide risk is significantly higher than overall suicide risk). Analyses were conducted using SEER*Stat software, version 8.3.6 (National Cancer Institute Cancer Statistics Branch) and Joinpoint software, version 4.7.0.0 (National Cancer Institute Cancer Statistics Branch). All statistical tests were 2-sided, and *P* < .05 was considered to be statistically significant.

## Results

SEER 18 included data on 5 362 782 persons with cancer diagnoses living in 635 counties. Most study participants were men (51.2%), White (72.2%), and older than 65 years (49.7%). Of those people diagnosed with cancer, 75.9% resided in the highest-income counties, and 5.6% resided in the lowest-income counties. In addition, 87.4% of people diagnosed with cancer lived in urban counties, and 12.6% lived in rural counties (Table 1). During 25 779 177 person-years of follow-up (median, 3.5; IQR, 0.9-8.3 years), 6357 persons died of suicide. Of 6357 deaths, 71.1% occurred in the highest-income counties and 84.3% occurred in urban counties. The expected number of suicide deaths for this population was 4510 (SMR, 1.41; 95% CI, 1.38-1.44; excess absolute risk, 0.72 per 10 000 persons).

**Table 1.** Baseline Characteristics by County Attribute, SEER 18, 2000-2016

Characteristic	No. (%)^a^					
	Annual median household income quartiles^b^				Rural/urban status^c^	
	1 (lowest)	2	3	4 (highest)	Rural	Urban
Total	299 268 (5.6)	297 706 (5.6)	696 914 (13.0)	4 067 783 (75.9)	676 543 (12.6)	4 685 117 (87.4)
Sex						
Male	161 370 (53.9)	158 721 (53.3)	364 491 (52.3)	2 062 816 (50.7)	362 705 (53.6)	2 384 685 (50.9)
Female	137 898 (46.1)	138 985 (46.7)	332 423 (47.7)	2 004 967 (49.3)	313 838 (46.4)	2 300 432 (49.1)
Race and ethnicity						
Hispanic	12 257 (4.1)	18 204 (6.1)	60 299 (8.7)	450 724 (11.1)	22 437 (3.3)	519 047 (11.1)
Non-Hispanic						
Asian/Pacific Islander	1057 (0.4)	1700 (0.6)	10 392 (1.5)	336 316 (8.3)	15 489 (2.3)	333 968 (7.1)
Black	57 143 (19.1)	45 969 (15.4)	77 279 (11.1)	398 655 (9.8)	56 561 (8.4)	522 485 (11.2)
White	225 431 (75.3)	229 053 (76.9)	544 895 (78.2)	2 869 591 (70.5)	575 968 (85.1)	3 292 999 (70.3)
Other^d^	3380 (1.1)	2780 (0.9)	4049 (0.6)	12 497 (0.3)	6088 (0.9)	16 618 (0.4)
Age, y						
<40	16 054 (5.4)	16 761 (5.6)	43 134 (6.2)	286 612 (7.0)	35 023 (5.2)	327 536 (7.0)
40-64	129 760 (43.4)	125 860 (42.3)	296 001 (42.5)	1 781 083 (43.8)	281 621 (41.6)	2 051 078 (43.8)
≥65	153 454 (51.3)	155 085 (52.1)	357 779 (51.3)	2 000 088 (49.2)	359 899 (53.2)	2 306 503 (49.2)

Abbreviations: SEER, Surveillance, Epidemiology, and End Results.

^a^ There are individuals in the SEER database missing county-level attribute information.

^b^ First quartile, $9330-$29 680; second quartile, $29 690-$33 850; third quartile, $33 860-$39 570; and fourth quartile, $39 580-$82 930.

^c^ Rural, nonmetropolitan counties (Rural/Urban Continuum codes 4-9); urban, metropolitan counties (Rural/Urban Continuum codes 1-3).

^d^ Non-Hispanic American Indian/Alaska Native and unknown.

### Suicide Risk by County Attribute

Increased risks of death from suicide among persons with a cancer diagnosis were observed among people living in low-income counties and among people living in rural counties (Table 2). Compared with people with cancer living in the highest-income counties (fourth income quartile: SMR, 1.30; 95% CI, 1.26-1.34), people living in the lowest-income counties had a significantly elevated risk for suicide death (first income quartile: SMR, 1.94; 95% CI, 1.76-2.13; second income quartile: SMR, 2.06; 95% CI, 1.88-2.25; third income quartile: SMR, 1.61; 95% CI, 1.51-1.72). People with cancer living in rural counties had a significantly increased risk of suicide (SMR, 1.81; 95% CI, 1.70-1.92) compared with people living in urban counties (SMR, 1.35; 95% CI, 1.32-1.39).

**Table 2.** Suicide Risk Among People With Cancer Diagnosis by County Attribute, SEER 18, 2000-2016

County attributes	Persons with cancer, No.	Person-years at risk	Deaths, No.		SMR, observed vs expected (95% CI)	EAR (per 10 000)
			Observed	Expected		
Income quartiles^a^						
First (lowest)	299 268	1 271 934	420	216.82	1.94 (1.76-2.13)	1.60
Second	297 706	1 320 449	479	232.94	2.06 (1.88-2.25)	1.86
Third	696 914	3 215 129	935	580.90	1.61 (1.51-1.72)	1.10
Fourth (highest)	4 067 783	19 965 934	4521	3478.15	1.30 (1.26-1.34)	0.52
Rural/urban status^b^						
Rural	676 543	3 039 873	1017	562.28	1.81 (1.70-1.92)	1.50
Urban	4 685 117	22 733 554	5338	3946.53	1.35 (1.32-1.39)	0.61

Abbreviations: EAR, excess absolute risk; SEER, Surveillance, Epidemiology, and End Results; SMR, standardized mortality ratio.

^a^ 1st quartile, $9330-$29 680; 2nd quartile, $29 690-$33 850; 3rd quartile, $33 860-$39 570; 4th quartile, $39 580-$82 930.

^b^ Rural, nonmetropolitan counties (Rural/Urban Continuum codes 4-9); Urban, metropolitan counties (Rural/Urban Continuum codes 1-3).

When further stratified by individual-level attributes, the observed patterning across county-level attributes was largely similar across sex, race and ethnicity, and age groups **(**eTable 1 in the Supplement). Although those aged 65 years and older had the largest disparities across county-level attributes (first quartile: SMR, 2.20; 95% CI, 1.93-2.49 vs fourth quartile: SMR, 1.43; 95% CI, 1.37-1.49), the disparity patterns were similar in all age groups. Men (first quartile: SMR, 2.09; 95% CI, 1.89-2.31 vs fourth quartile: SMR, 1.32; 95% CI, 1.28-1.36) had a larger discrepancy across county-level income than women (first quartile: SMR, 1.40; 95% CI, 1.05-1.82 vs fourth quartile: SMR, 1.22; 95% CI, 1.14-1.31), although with similar patterning. Among White individuals, the SMR was significantly higher in the lowest-income counties (SMR, 2.01; 95% CI, 1.82-2.21) than in the highest-income counties (SMR, 1.42; 95% CI, 1.37-1.46). These patterns were also observed when comparing rural and urban counties. However, among Asian/Pacific Islander individuals with cancer, the elevated risk of suicide death did not differ across county-level income quartiles. Asian/Pacific Islander individuals with cancer who lived in the highest-income counties had a nearly 2-fold increased risk of suicide death (SMR, 1.95; 95% CI, 1.72-2.22) compared with the general population, which was similar to the excess risk of suicide death among Asian/Pacific Islander individuals living in lower-income quartiles. In contrast to the other racial and ethnic groups, Hispanic people with cancer living in the lowest-income counties did not have a significantly increased risk of suicide death (first quartile: SMR, 1.26; 95% CI, 0.69-2.11). Hispanic people with cancer living in the highest-income counties had a significantly lower risk of suicide death (fourth quartile: SMR, 0.63; 95% CI, 0.55-0.71) than the general population.

### Latency-Specific Suicide Risk by County Attribute

**Figure 1.**
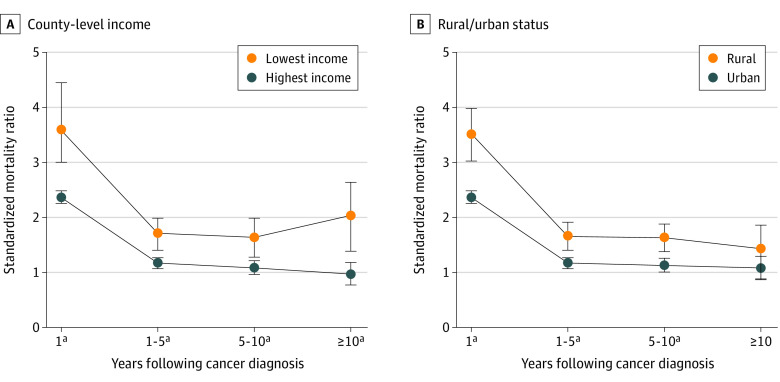
Latency-Specific Suicide Risk by County Attribute Standardized mortality ratios over latency periods following cancer diagnosis by county-level income quartile (A) and rural vs urban status (B). Error bars indicate 95% CIs. ^a^Statistically significant finding.

Regardless of county-level income quartile or rural or urban status, the risk of death by suicide was highest within the first year after cancer diagnosis (Figure 1). Among people with cancer in all county groups, the risk of suicide death decreased beyond the first year following cancer diagnosis. By 10 or more years after cancer diagnosis, those living in the highest-income counties did not have an increased risk of suicide death, nor were significant differences observed in the risk of suicide death by rural or urban status. However, people with cancer living in the lowest-income counties had a persistently increased risk of suicide death: by 10 or more years following cancer diagnosis, their SMR was 1.83 (95% CI, 1.31-2.48). Complete SMRs by latency for all county-level attributes are reported in eTable 2 in the Supplement.

### Temporal Trends in Suicide Risk After Diagnosis

**Figure 2.**
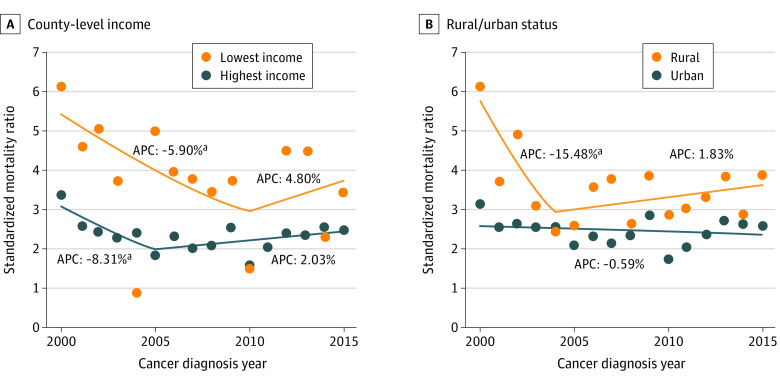
First-Year Suicide Risk During Diagnosis Years by County Attribute Annual percentage changes (APCs) of standardized mortality ratios (SMRs) over cancer diagnosis years by county-level income quartile (A) and rural vs urban status (B). ^a^Statistically significant finding.

Temporal trends in suicide risk during the year following cancer diagnosis were different when SMRs were stratified by county groups (Figure 2; eTable 3 in the Supplement). Among people with cancer living in the lowest-income counties, the first-year suicide SMR declined from 2000 to 2010 (APC, −5.90%; 95% CI, −12.92% to −0.31%) and then plateaued at 2010-2015 (APC, 4.80%; 95% CI, −19.97% to 37.24%). Similarly, among people with cancer living in the highest-income counties, the first-year suicide SMR declined from 2000 to 2005 (APC, −8.31%; 95% CI, −15.75% to −0.21%), and there was no statistically significant change in 2005-2015 (APC_,_ 2.03%; 95% CI, −0.97% to 5.13%). Among people with cancer living in rural counties, the first-year suicide SMR decreased from 2000 to 2004 (APC, −15.48%; 95% CI, −28.45% to −0.17%), followed by a plateau from 2004 to 2015 (APC, 1.83%; 95% CI, −1.98% to 5.79%). Among people with cancer living in urban counties, the first-year suicide SMR did not significantly change from 2000 to 2015 (APC_,_ −0.59%; 95% CI, −2.20% to 1.05%).

### Suicide Risk by Other County-Level Factors

Suicide rates among people diagnosed with cancer were higher in counties with lower educational attainment, higher unemployment rates, and a higher percentage of Black residents (eTable 4 and eTable 5 in the Supplement). Also, when we cross-stratified by income and rural or urban status, suicide risk among people diagnosed with cancer was significantly higher in rural counties compared with urban counties, but only among counties within the highest-income quartile (SMR, 1.58; 95% CI, 1.35-1.84 vs 1.29; 95% CI, 1.25-1.33) (eTable 6 in the Supplement). When we examined suicide risk among people diagnosed with cancer, stratified jointly by the percentage of Black residents and income quartiles, the highest suicide risk was observed in counties in the lowest income quartile with more than 50% Black residents (SMR, 2.47; 95% CI, 1.84-3.25). When we examined suicide risk by percentage of Black residents and rural or urban status jointly, rural counties with more than 50% Black residents had the highest suicide rate (SMR, 3.51; 95% CI, 2.12-5.49) (eTables 7-9 in the Supplement).

## Discussion

In this study, we noted significant disparities in suicide mortality and its temporal patterning between rural vs urban counties and high- vs low-income counties. We found higher suicide mortality following cancer diagnosis among people living in lower-income and rural counties. These disparities in suicide deaths following cancer diagnosis are consistent with the same disparities observed in the general population.^18,19^ However, we noted that Asian/Pacific Islander individuals with cancer tended to have consistent suicide risk across county-level strata. It is possible that cultural factors may be involved in this result. Numerous studies have demonstrated that social stigma, shame, and “saving face” often prevent Asian and Asian American individuals from seeking mental health care.^20,21,22,23,24^ Meanwhile, the risk of suicide following cancer diagnosis among Hispanic persons was no different or potentially even lower compared with the general population, with a 37% lower risk in the highest-income counties. This finding may be partially explained by the Hispanic health paradox, a well-established epidemiologic finding that Hispanic people tend to have lower mortality rates compared with non-Hispanic people.^25^ In this study, White people, men, and those aged 65 years or older exhibited the greatest disparities by county-level income and rural status.

We found the highest suicide risks within 1 year immediately after cancer diagnosis regardless of county-level attribute, consistent with previously reported results.^2,3,5^ However, among people living in the lowest-income counties, suicide mortality risk decreased during 1 to 10 years following cancer diagnosis and increased again to a rate nearly twice that of the general population after more than 10 years following cancer diagnosis. A higher risk of financial toxicity may partially explain this finding in low-income individuals with cancer^26^; mental distress from financial toxicity might worsen during the course of cancer survivorship. This finding warrants further research, especially given the high rates of financial hardship among cancer survivors.^27^

The assessment of area-level social vulnerability as a social determinant of health captures conditions that affect all individuals living in the same area. People living in low-income counties had an increased suicide risk following cancer diagnosis compared with those living in high-income counties, potentially owing to the fragility of the health and social services safety net and the lack of comprehensive cancer survivorship care in less-affluent neighborhoods. People living in low-income counties had a sustained risk of suicide even after 10 or more years following cancer diagnosis. Previous studies have shown that low-income counties have the fewest mental health professionals per capita and have higher levels of unmet mental health service needs compared with high-income counties.^14,15^ Rural neighborhoods also lack mental health care facilities and professionals, and their residents experience access barriers (eg, travel time) more often than residents in urban neighborhoods.^14,15^ Disparities in mental health between cancer survivors living in rural vs urban counties have been reported.^28,29^ The American Society of Clinical Oncology recommends routine screening for depression at regular intervals and significant points in cancer treatment and survivorship care.^30^ Our results support the need for improved access to mental health care for patients with cancer and survivors, particularly those living in low-income and rural areas.

We also found that low-income counties had a smaller decrease in first-year suicide SMR temporal trends compared with high-income counties. Assessing temporal trends in the first-year risk of suicide allowed us to compare changes free of bias from risk differences by latency after cancer diagnosis. Although urban counties did not show a significant change in SMRs over calendar years, rural counties experienced a significant decrease until 2004, followed by a plateau. Decreasing patterns over the years in all cancer-related suicide were also reported by Han et al.^31^ These patterns could potentially be explained by increased use of comprehensive cancer care, such as psychological, palliative, and hospice care,^31,32,33^ consistent with prevailing efforts to improve survivorship care and promulgate guideline-consistent care among oncology professionals.^34,35^ Notably, cancer survival rates concurrently improved during the study period; thus, improvement in early detection and treatment may also have contributed to decreasing cancer-related suicide.^36^ However, these decreasing changes plateaued in rural counties, warranting further attention. Given the increasing suicide rates in the general population,^31,37,38^ it is possible that our findings may be due to a real shift toward increasing suicide rates among persons with cancer. If the shifts continue in the county groups, there may be more significant discrepancies between low-income counties and high-income counties, as well as between rural counties and urban counties.

In additional analyses, we found that suicide risk among people diagnosed with cancer differed little according to rurality among counties in the lowest-income quartiles, while the highest-income counties showed significant rural vs urban disparities. This finding may indicate that people diagnosed with cancer living in low-income areas lack access to mental health care regardless of the degree of rurality. Also, we analyzed suicide rates by the county-level percentage of Black residents, given that area-level affluence has historically been associated with the racial and ethnic make-up (eg, owing to residential segregation and other fundamental mechanisms of structural discrimination and poor health).^39,40^ As an ecologic factor, we found that county-level racial composition had a statistically significant association with suicide mortality rates. This disparity was especially notable within rural counties. This result suggests that racial residential segregation may be more closely associated with poorer health care access and overall quality of life in rural neighborhoods compared with urban neighborhoods.^41^

### Strengths and Limitations

The study has limitations. First, there could be misclassification in the cause of death because suicide is often difficult to distinguish from accidental injury or homicide. Owing to limited literature regarding the misclassification of suicide in the registry, we are not in a position to determine the direction of possible bias. Second, the county-level characteristics are measured at the level of the county. Within large and heterogeneous counties, data on variables such as median household income might convey less information compared with small, homogeneous counties. Third, our analysis explored a limited number of National Cancer Institute–supported county characteristics. Future studies should consider investigating other county-level socioeconomic characteristics and their role in explaining suicide mortality among people diagnosed with cancer. Despite these limitations, our study adds to the literature by assessing suicide mortality after cancer diagnosis according to multiple ecological indicators of social vulnerability. We also identify a prolonged risk of suicide mortality among people diagnosed with cancer living in low-income counties. The findings are generalizable to the US population, given the nationally representative nature of the cancer registry data sets.

## Conclusions

In the US, although suicide risks are overall higher among people with cancer compared with people in the general population, people with cancer who live in low-income and rural counties appear to be at greater risk of suicide, with much-prolonged suicide risk in low-income counties. Our findings support efforts to provide increased preventive mental health services, especially for those living in low-income and rural areas. Although barriers to mental health care for patients with cancer or cancer survivors in these areas likely reflect a complex web of access problems,^42^ the increased use of telemedicine for medical and mental health care could be a possible way to increase access to care.^43^

## Notes

Supplement. **eTable 1.** Suicide Among Persons With Cancer Diagnosis, Stratified by County-Level Income and Rural vs Urban Status and by Individual Sociodemographic Factors, SEER 18, 2000-2016 **eTable 2.** Standardized Morality Ratios (SMRs) by Latency Year, Stratified by County-Level Income and Rural vs Urban Status **eTable 3.** Temporal Trends in First 1-Year Suicide Standardized Morality Ratios (SMRs), Stratified by County-Level Income and Rural vs Urban Status, SEER 18, 2000-2016 **eTable 4.** Suicide Risk Among Persons With Cancer Diagnosis, Stratified by County-Level Educational Attainment, SEER 18, 2000-2016 **eTable 5.** Suicide Risk Among Persons With Cancer Diagnosis, Stratified by County-Level Unemployment, SEER 18, 2000-2016 **eTable 6.** Suicide Risk Among Persons With Cancer Diagnosis, Cross-Stratified by County-Level Income and Rural vs Urban Status, SEER 18, 2000-2016 **eTable 7.** Suicide Risk Among Persons With Cancer Diagnosis, Stratified by County-Level Percentage of Black Residents, SEER 18, 2000-2016 **eTable 8.** Suicide Risk Among Persons With Cancer Diagnosis, Cross-Stratified by County-Level Income and Percentage of Black Residents, SEER 18, 2000-2016 **eTable 9.** Suicide Risk Among Persons With Cancer Diagnosis, Cross-Stratified by County-Level Rural vs Urban Status and Percentage of Black Residents, SEER 18, 2000-2016
