# Supplementary material for: Analysis of Suicide After Cancer Diagnosis by US County-Level Income and Rural vs Urban Designation, 2000-2016
Source: JAMA Netw Open. 2021 Oct 19;4(10):e2129913. doi: 10.1001/jamanetworkopen.2021.29913 (PMC8527360; doi:10.1001/jamanetworkopen.2021.29913)
Supplement: Supplement. — eTable 1. Suicide Among Persons With Cancer Diagnosis, Stratified by County-Level Income and Rural vs Urban Status and by Individual Sociodemographic Factors, SEER 18, 2000-2016 eTable 2. Standardized Morality Ratios (SMRs) by Latency Year, Stratified by County-Level Income and Rural vs Urban Status eTable 3. Temporal Trends in First 1-Year Suicide Standardized Morality Ratios (SMRs), Stratified by County-Level Income and Rural vs Urban Status, SEER 18, 2000-2016 eTable 4. Suicide Risk Among Persons With Cancer Diagnosis, Stratified by County-Level Educational Attainment, SEER 18, 2000-2016 eTable 5. Suicide Risk Among Persons With Cancer Diagnosis, Stratified by County-Level Unemployment, SEER 18, 2000-2016 eTable 6. Suicide Risk Among Persons With Cancer Diagnosis, Cross-Stratified by County-Level Income and Rural vs Urban Status, SEER 18, 2000-2016 eTable 7. Suicide Risk Among Persons With Cancer Diagnosis, Stratified by County-Level Percentage of Black Residents, SEER 18, 2000-2016 eTable 8. Suicide Risk Among Persons With Cancer Diagnosis, Cross-Stratified by County-Level Income and Percentage of Black Residents, SEER 18, 2000-2016 eTable 9. Suicide Risk Among Persons With Cancer Diagnosis, Cross-Stratified by County-Level Rural vs Urban Status and Percentage of Black Residents, SEER 18, 2000-2016 [file jamanetwopen-e2129913-s001.pdf]

Supplementary Online Content

Suk R, Hong YR, Wasserman RM, et al. Analysis of suicide after cancer diagnosis by US county-level income and rural vs urban designation, 2000-2016. *JAMA Netw Open*. 2021;4(10):e2129913. doi:10.1001/jamanetworkopen.2021.29913

eTable 1. Suicide Among Persons With Cancer Diagnosis, Stratified by County-Level Income and Rural vs Urban Status and by Individual Sociodemographic Factors, SEER 18, 2000-2016

eTable 2. Standardized Morality Ratios (SMRs) by Latency Year, Stratified by County-Level Income and Rural vs Urban Status

eTable 3. Temporal Trends in First 1-Year Suicide Standardized Morality Ratios (SMRs), Stratified by County-Level Income and Rural vs Urban Status, SEER 18, 2000-2016

eTable 4. Suicide Risk Among Persons With Cancer Diagnosis, Stratified by County-Level Educational Attainment, SEER 18, 2000-2016

eTable 5. Suicide Risk Among Persons With Cancer Diagnosis, Stratified by County-Level Unemployment, SEER 18, 2000-2016

eTable 6. Suicide Risk Among Persons With Cancer Diagnosis, Cross-Stratified by County-Level Income and Rural vs Urban Status, SEER 18, 2000-2016

eTable 7. Suicide Risk Among Persons With Cancer Diagnosis, Stratified by County-Level Percentage of Black Residents, SEER 18, 2000-2016

eTable 8. Suicide Risk Among Persons With Cancer Diagnosis, Cross-Stratified by County-Level Income and Percentage of Black Residents, SEER 18, 2000-2016

eTable 9. Suicide Risk Among Persons With Cancer Diagnosis, Cross-Stratified by County-Level Rural vs Urban Status and Percentage of Black Residents, SEER 18, 2000-2016

This supplementary material has been provided by the authors to give readers additional information about their work.

© 2021 Suk R et al. *JAMA Network Open.*

eTable 1. Suicide Among Persons With Cancer Diagnosis, Stratified by County-Level Income and Rural vs Urban Status and by Individual Sociodemographic Factors, SEER 18, 2000-2016

	Observed Deaths, No.	Expected Deaths, No.	SMR (O/E) (95% CI)	EAR (per 10,000)	Persons with Cancer, No.	Person Years at Risk
1st Income Quartile						
Sex						
Male	401	192	2,09 (1.89-2.31)	3.00	167,184	698,107
Female	55	39	1.40 (1.05-1.82)	0.24	144,846	645,908
Race/Ethnicity						
NH White	413	205	2.01 (1.82-2.21)	1.99	238,184	1,042,782
NH Black	26	13	1.97 (1.29-2.88)	0.57	55,819	225,944
NH API	1	1	1.94 (0.05-10.81)	0.75	1,648	6,447
Hispanic	14	11	1.26 (0.69-2.11)	0.50	13,572	57,365
NH Other	2	1	2.17 (0.26-7.84)	0.94	2,807	11,477
Age						
<40	21	13	1.59 (0.98-2.43)	0.72	17,294	108,361
40-64	194	108	1.79 (1.55-2.06)	1.27	136,412	672,039
≥65	241	110	2.20 (1.93-2.49)	2.33	158,324	563,616
2nd Income Quartile						
Sex						
Male	389	201	1.94 (1.75-2.14)	2.65	163,531	709,587
Female	69	41	1.69 (1.32-2.14)	0.43	144,972	657,200
Race/Ethnicity						
NH White	414	214	1.94 (1.76-2.13)	1.86	238,605	1,078,012
NH Black	23	11	2.11 (1.34-3.17)	0.65	45,099	186,445
NH API	2	1	2.05 (0.25-7.40)	0.78	3,380	13,065
Hispanic	17	15	1.13 (0.66-1.81)	0.25	18,893	78,627
NH Other	2	1	2.30 (0.28-8.30)	1.06	2,526	10,638
Age						
<40	14	14	1.00 (0.54-1.67)	0.00	18,027	112,711

© 2021 Suk R et al. *JAMA Network Open.*

40-64	185	109	1.69 (1.46-1.95)	1.14	131,091	666,650
≥65	259	118	2.19 (1.93-2.48)	2.40	159,385	587,426
3rd Income Quartile						
Sex						
Male	795	471	1.69 (1.57-1.81)	2.00	358,837	1,621,410
Female	131	99	1.32 (1.10-1.56)	0.20	328,755	1,546,760
Race/Ethnicity						
NH White	858	501	1.71 (1.60-1.83)	1.41	537,403	2,537,662
NH Black	23	18	1.27 (0.81-1.91)	0.16	74,647	311,385
NH API	9	4	2.14 (0.98-4.07)	0.92	13,033	52,021
Hispanic	32	46	0.70 (0.48-0.99)	-0.55	58,336	249,437
NH Other	4	1	2.84 (0.77-7.28)	1.47	4,173	17,665
Age						
<40	58	35	1.67 (1.27-2.16)	0.84	43,387	275,377
40-64	362	263	1.38 (1.24-1.53)	0.63	293,687	1,562,138
≥65	506	273	1.85 (1.70-2.02)	1.75	350,518	1,330,654
4th Income Quartile						
Sex						
Male	3,740	2,832	1.32 (1.28-1.36)	0.91	2,057,702	10,028,759
Female	774	633	1.22 (1.14-1.31)	0.14	1,995,534	9,864,018
Race/Ethnicity						
NH White	3,873	2,868	1.35 (1.31-1.39)	0.69	2,854,576	14,525,426
NH Black	152	106	1.44 (1.22-1.68)	0.26	403,453	1,787,791
NH API	240	123	1.95 (1.72-2.22)	0.78	331,394	1,505,271
Hispanic	228	364	0.63 (0.55-0.71)	-0.67	450,625	2,017,192
NH Other	21	5	4.52 (2.80-6.91)	2.86	13,188	57,096
Age						
<40	251	236	1.06 (0.94-1.20)	0.08	283,839	1,853,730
40-64	2,003	1,644	1.22 (1.17-1.27)	0.36	1,771,393	10,082,680
≥65	2,260	1,585	1.43 (1.37-1.49)	0.85	1,998,004	7,956,367
Rural						
Sex						

© 2021 Suk R et al. *JAMA Network Open.*

Male	929	507	1.83 (1.72-1.95)	2.42	394,339	1,742,257
Female	149	105	1.42 (1.20-1.67)	0.27	350,448	1,630,994
Race/Ethnicity						
NH White	986	559	1.76 (1.66-1.88)	1.51	610,993	2,818,237
NH Black	28	16	1.77 (1.17-2.55)	0.45	66,483	267,525
NH API	32	11	2.80 (1.92-3.95)	1.51	30,921	135,960
Hispanic	26	24	1.08 (0.71-1.58)	0.15	30,889	128,585
NH Other	6	2	3.25 (1.19-7.07)	1.81	5,501	22,945
Age						
<40	49	35	1.42 (1.05-1.88)	0.54	42,103	269,547
40-64	451	279	1.62 (1.47-1.77)	1.05	314,087	1,643,195
≥65	578	299	1.93 (1.78-2.10)	1.91	388,597	1,460,510
Urban						
Sex						
Male	4,396	3,188	1.38 (1.34-1.42)	1.07	2,352,914	11,315,604
Female	880	708	1.24 (1.16-1.33)	0.16	2,263,659	11,082,892
Race/Ethnicity						
NH White	4,572	3,230	1.42 (1.37-1.46)	0.82	3,257,774	16,365,645
NH Black	196	132	1.48 (1.28-1.71)	0.28	512,535	2,244,041
NH API	220	117	1.88 (1.64-2.15)	0.71	318,534	1,440,843
Hispanic	265	411	0.64 (0.57-0.73)	-0.64	510,537	2,274,036
NH Other	23	6	3.83 (2.43-5.75)	2.30	17,193	73,930
Age						
<40	295	263	1.12 (1.00-1.26)	0.15	320,443	2,080,631
40-64	2,293	1,846	1.24 (1.19-1.29)	0.39	2,018,496	11,340,311
≥65	2,688	1,787	1.50 (1.45-1.56)	1.00	2,277,634	8,977,553

SEER, Surveillance, Epidemiology, and End Results;
No., number; SMR, standardized mortality ratio; O/E, observed/expected; CI, confidence interval; EAR, excess absolute risk
1st quartile, $9,330-$29,680 (178 counties); 2nd quartile, $29,690-$33,850 (116 counties); 3rd quartile, $33,860-$39,570 (152 counties); 4th quartile, $39,580-$82,930 (166 counties)
Rural, Non-metropolitan counties (Rural vs Urban Continuum codes 4-9; 366 counties); Urban, Metropolitan counties (Rural vs Urban Continuum codes 1-3; 246 counties)
NH, non-Hispanic; API, Asian/Pacific Islander

© 2021 Suk R et al. *JAMA Network Open.*

eTable 2. Standardized Morality Ratios (SMRs) by Latency Year, Stratified by County-Level Income and Rural vs Urban Status

County Attributes	Observed Deaths, No.	Expected Deaths, No.	SMR (O/E) (95% CI)	EAR (per 10,000)	Persons with Cancer, No.	Person Years at Risk
Income quartiles						
1st (lowest income)						
<1 year	140	39	3.60 (3.03-4.25)	4.30	299,268	234,984
1-5 years	152	95	1.60 (1.36-1.88)	1.01	201,190	566,079
5-10 years	87	40	1.44 (1.15-1.77)	0.77	101,967	345,535
≥10 years	41	22	1.83 (1.31-2.48)	1.48	42,027	125,336
2nd						
<1 year	143	40	3.54 (2.98-4.17)	4.32	297,706	237,552
1-5 years	184	102	1.81 (1.56-2.09)	1.41	205,507	586,445
5-10 years	116	66	1.76 (1.45-2.11)	1.38	106,547	363,784
≥10 years	36	25	1.43 (1.00-1.99)	0.82	44,361	132,669
3rd						
<1 year	384	98	2.91 (2.58-3.26)	3.31	696,914	563,520
1-5 years	357	251	1.42 (1.28-1.58)	0.75	491,489	1,419,750
5-10 years	221	167	1.32 (1.15-1.51)	0.60	261,016	897,554
≥10 years	73	65	1.12 (0.88-1.41)	0.24	110,710	334,304
4th (highest income)						
<1 year	1,308	559	2.34 (2.22-2.47)	2.23	4,067,783	3,361,329
1-5 years	1,735	1,486	1.17 (1.11-1.22)	0.29	2,967,466	8,737,301
5-10 years	1,065	1,022	1.04 (0.98-1.11)	0.08	1,631,719	5,691,190
≥10 years	413	411	1.00 (0.91-1.11)	0.01	714,873	2,176,114
Rural vs Urban status						
Rural						
<1 year	337	97	3.48 (3.11-3.87)	4.43	676,543	541,970

© 2021 Suk R et al. *JAMA Network Open.*

1-5 years	373	244	1.53 (1.38-1.69)	0.96	469,786	1,345,397
5-10 years	232	160	1.45 (1.27-1.65)	0.85	245,823	843,257
≥10 years	75	61	1.23 (0.97-1.55)	0.46	103,453	309,249
Urban						
<1 year	1.,538	639	2.41 (2.29-2.53)	2.33	4,685,117	3,855,409
1-5 years	2,055	1,689	1.22 (1.16-1.27)	0.37	3,395,862	9,964,170
5-10 years	1,257	1,155	1.09 (1.03-1.15)	0.16	1,855,425	6,454,802
≥10 years	488	463	1.05 (0.96-1.15)	0.10	808,518	2,459,173

SEER, Surveillance, Epidemiology, and End Results;
No., number; SMR, standardized mortality ratio; O/E, observed/expected; CI, confidence interval; EAR, excess absolute risk
1st quartile, $9,330-$29,680 (178 counties); 2nd quartile, $29,690-$33,850 (116 counties); 3rd quartile, $33,860-$39,570 (152 counties); 4th quartile, $39,580-$82,930 (166 counties)
Rural, Non-metropolitan counties (Rural vs Urban Continuum codes 4-9; 366 counties); Urban, Metropolitan counties (Rural vs Urban Continuum codes 1-3; 246 counties)

© 2021 Suk R et al. *JAMA Network Open.*

eTable 3. Temporal Trends in First 1-Year Suicide Standardized Morality Ratios (SMRs), Stratified by County-Level Income and Rural vs Urban Status, SEER 18, 2000-2016

	Trends		
	**Segments**		**Overall (2000-2015)**
	**Year**	**APC (95% CI)**	**AAPC (95% CI)**
Income quartiles			
1st (lowest income)	2000-2010<br>2010-2015	-5.90 (-14.92 to -0.31)<br>4.80 (-19.97 to 37.24)	-2.46 (-11.06 to 6.98)
2nd	2000-2015	0.27 (-3.99 to 4.71)	0.27 (-3.99 to 4.71)
3rd	2000-2005<br>2005-2015	-4.64 (-9.25 to -0.03)<br>1.64 (-5.78 to 9.64)	-0.50 (-8.03 to 7.65)
4th (highest income)	2000-2005<br>2005-2015	-8.31 (-15.75 to -0.21)<br>2.03 (-0.97 to 5.13)	-1.54 (-4.52 to 1.54)
Rural vs Urban status			
Rural	2000-2004<br>2004-2015	-15.48 (-28.45 to -0.17)<br>1.83 (-1.98 to 5.79)	-3.11 (-7.53 to 1.53)
Urban	2000-2015	-0.59 (-2.20 to 1.05)	-0.59 (-2.20 to 1.05)

SEER, Surveillance, Epidemiology, and End Results;
SMR, standardized mortality ratio; APC, annual percentage chance; AAPC, average annual percentage change; CI, confidence interval;
1st quartile, \$9,330-\$29,680 (178 counties); 2nd quartile, \$29,690-\$33,850 (116 counties); 3rd quartile, \$33,860-\$39,570 (152 counties); 4th quartile, \$39,580-\$82,930 (166 counties)
Rural, Non-metropolitan counties (Rural vs Urban Continuum codes 4-9; 366 counties); Urban, Metropolitan counties (Rural vs Urban Continuum codes 1-3; 246 counties)

© 2021 Suk R et al. *JAMA Network Open.*

eTable 4. Suicide Risk Among Persons With Cancer Diagnosis, Stratified by County-Level Educational Attainment, SEER 18, 2000-2016

County Attributes	Observed Deaths, No.	Expected Deaths, No.	SMR (O/E) (95% CI)	EAR (per 10,000)	Persons with Cancer, No.	Person Years at Risk
% of less than high-school quartiles						
1st (lowest % of less than high-school)	1,942	1,542	1.26 (1.20-1.32)	0.47	1,673,993	8,533,932
2nd	2,008	1,430	1.40 (1.34-1.47)	0.71	1,681,292	8,174,163
3rd	1,050	343	1.63 (1.54-1.74)	1.06	840,799	3,833,998
4th (highest % of less than high-school)	1,355	894	1.52 (1.44-1.60)	0.88	1,165,587	5,231,353

SEER, Surveillance, Epidemiology, and End Results;
No., number; SMR, standardized mortality ratio; O/E, observed/expected; CI, confidence interval; EAR, excess absolute risk
1st quartile, 3.04%-15.91% (144 counties); 2nd quartile, 15.92%-20.76% (114 counties); 3rd quartile, 20.77%-28.67% (120 counties); 4th quartile, 28.68%-65.30% (235 counties)

© 2021 Suk R et al. *JAMA Network Open.*

eTable 5. Suicide Risk Among Persons With Cancer Diagnosis, Stratified by County-Level Unemployment, SEER 18, 2000-2016

County Attributes	Observed Deaths, No.	Expected Deaths, No.	SMR (O/E) (95% CI)	EAR (per 10,000)	Persons with Cancer, No.	Person Years at Risk
Unemployment rate quartiles						
1st (lowest unemployment)	872	711	1.23 (1.15-1.31)	0.41	779,188	3,906,391
2nd	1,629	1,273	1.28 (1.22-1.34)	0.51	1,401,798	6,996,138
3rd	1,686	1,104	1.53 (1.45-1.60)	0.91	1,334,647	6,412,408
4th (highest unemployment)	2,168	1,421	1.53 (1.46-1.59)	0.88	1,846,038	8,458,509

SEER, Surveillance, Epidemiology, and End Results;
No., number; SMR, standardized mortality ratio; O/E, observed/expected; CI, confidence interval; EAR, excess absolute risk
1st quartile, 0.00%-3.97% (132 counties); 2nd quartile, 3.98%-5.35% (135 counties); 3rd quartile, 5.36%-6.95% (147 counties); 4th quartile, 6.96%-41.67% (199 counties)

© 2021 Suk R et al. *JAMA Network Open.*

eTable 6. Suicide Risk Among Persons With Cancer Diagnosis, Cross-Stratified by County-Level Income and Rural vs Urban Status, SEER 18, 2000-2016

County Attributes	Observed Deaths, No.	Expected Deaths, No.	SMR (O/E) (95% CI)	EAR (per 10,000)	Persons with Cancer, No.	Person Years at Risk
Income quartiles and rural/urban status						
1st (lowest income)						
Rural	365	190	1.92 (1.73-2.13)	1.64	252,842	1,068,164
Urban	55	27	2.02 (1.52-2.63)	1.36	46,426	203,770
2nd						
Rural	240	120	2.01 (1.76-2.28)	1.87	144,630	642,786
Urban	239	113	2.11 (1.85-2.39)	1.85	153,076	677,664
3rd						
Rural	249	150	1.66 (1.46-1.88)	1.29	163,567	766,324
Urban	686	431	1.59 (1.47-1.72)	1.04	533,347	2,448,805
4th (highest income)						
Rural	163	103	1.58 (1.35-1.84)	1.06	155,504	562,600
Urban	4,358	3,375	1.29 (1.25-1.33)	0.51	3,952,268	19,403,316

SEER, Surveillance, Epidemiology, and End Results;
No., number; SMR, standardized mortality ratio; O/E, observed/expected; CI, confidence interval; EAR, excess absolute risk
1st quartile, $9,330-$29,680; 2nd quartile, $29,690-$33,850; 3rd quartile, $33,860-$39,570; 4th quartile, $39,580-$82,930
Rural, Non-metropolitan counties (Rural vs Urban Continuum codes 4-9); Urban, Metropolitan counties (Rural vs Urban Continuum codes 1-3)
1st income quartile & rural: 163 counties; 1st income quartile & urban: 15 counties; 2nd income quartile & rural: 83 counties; 2nd income quartile & urban: 33 counties; 3rd income quartile & rural: 89 counties; 3rd income quarlile & urban: 63 counties; 4th income quartile & rural: 31 counties; 4th income quartile & urban: 135 counties

© 2021 Suk R et al. *JAMA Network Open.*

eTable 7. Suicide Risk Among Persons With Cancer Diagnosis, Stratified by County-Level Percentage of Black Residents, SEER 18, 2000-2016

County Attributes	Observed Deaths, No.	Expected Deaths, No.	SMR (O/E) (95% CI)	EAR (per 10,000)	Persons with Cancer, No.	Person Years at Risk
% of Black population						
0%~<12.5%	4,865	3,382	1.44 (1.40-1.48)	0.79	3,864,341	18,754,202
12.5%~<30.0%	884	738	1.20 (1.12-1.28)	0.34	907,600	4,322,317
30.0%~<50.0%	490	325	1.51 (1.38-1.65)	0.76	476,991	2,181,654
≥50.0%	116	64	1.82 (1.51-2.19)	1.02	112,739	515,273

SEER, Surveillance, Epidemiology, and End Results;
No., number; SMR, standardized mortality ratio; O/E, observed/expected; CI, confidence interval; EAR, excess absolute risk
0-12.5% Black population: 413 counties; 12.5-30% Black population: 94 counties; 30-50% Black population: 79 coutines; >50% Black population: 26 counties

© 2021 Suk R et al. *JAMA Network Open.*

eTable 8. Suicide Risk Among Persons With Cancer Diagnosis, Cross-Stratified by County-Level Income and Percentage of Black Residents, SEER 18, 2000-2016

County Attributes	Observed Deaths, No.	Expected Deaths, No.	SMR (O/E) (95% CI)	EAR (per 10,000)	Persons with Cancer, No.	Person Years at Risk
Income quartiles and % of Black population						
1st (lowest income)						
0%~<12.5% Black pop.	220	113	1.95 (1.70-2.22)	1.80	139,446	592,898
12.5%~<30.0%	79	43	1.86 (1.47-2.31)	1.46	58,779	250,177
30.0%~<50.0%	70	41	1.73 (1.35-2.18)	1.14	61,563	258,495
≥50.0%	51	21	2.47 (1.84-3.25)	1.78	39,480	170,363
2nd						
0%~<12.5% Black pop.	297	144	2.07 (1.84-2.32)	2.07	166,346	741,741
12.5%~<30.0%	53	22	2.42 (1.81-3.16)	2.45	29,624	126,774
30.0%~<50.0%	103	55	1.86 (1.52-2.26)	1.34	80,164	356,203
≥50.0%	26	12	2.16 (1.41-3.16)	1.46	21,572	95,731
3rd						
0%~<12.5% Black pop.	636	383	1.66 (1.53-1.79)	1.26	431,450	2,010,741
12.5%~<30.0%	190	136	1.40 (1.20-1.61)	0.68	173,755	790,704
30.0%~<50.0%	109	62	1.77 (1.45-2.13)	1.14	91,709	413,684
≥50.0%	0	0	0	0	0	0
4th (highest income)						
0%~<12.5% Black pop.	3,712	2,743	1.35 (1.31-1.40)	0.63	3,127,099	15,408,822
12.5%~<30.0%	562	537	1.05 (0.96-1.14)	0.08	645,442	3,154,662
30.0%~<50.0%	208	167	1.24 (1.08-1.42)	0.35	243,555	1,153,271
≥50.0%	39	31	1.26 (0.90-1.72)	0.32	51,687	249,179

SEER, Surveillance, Epidemiology, and End Results;
No., number; SMR, standardized mortality ratio; O/E, observed/expected; CI, confidence interval; EAR, excess absolute risk; pop., population
1st quartile, $9,330-$29,680; 2nd quartile, $29,690-$33,850; 3rd quartile, $33,860-$39,570; 4th quartile, $39,580-$82,930
1st income quartile & 0-12.5% Black population: 90 counties; 1st income quartile & 12.5-30% Black population: 29 counties; 1st income quartile & 30-50% Black population: 40 counties; 1st income quartile & >50% Black population: 19 counties; 2nd income quartile & 0-12.5% Black population: 75 counties; 2nd income quartile & 12.5-30% Black population: 15 counties; 2nd income quartile & 30-50% Black population: 22 counties; 2nd income quartile & >50% Black population: 4 counties; 3rd income quartile & 0-12.5% Black population: 123 counties; 3rd income quartile & 12.5-30% Black population: 15 counties; 3rd income quartile & 30-50% Black population: 14 counties; 3rd income quartile & >50% Black population: 0; 4th income quartile & 0-12.5% Black population: 126 counties; 4th income

© 2021 Suk R et al. *JAMA Network Open.*

quartile & 12.5-30% Black population: 35 counties; 4th income quartile & 30-50% Black population: 3 counties; 4th income quartile & >50% Black population: 3 counties

© 2021 Suk R et al. *JAMA Network Open.*

eTable 9. Suicide Risk Among Persons With Cancer Diagnosis, Cross-Stratified by County-Level Rural vs Urban Status and Percentage of Black Residents, SEER 18, 2000-2016

County Attributes	Observed Deaths, No.	Expected Deaths, No.	SMR (O/E) (95% CI)	EAR (per 10,000)	Persons with Cancer, No.	Person Years at Risk
Rural/urban status and % of Black population						
Rural						
0%~<12.5% Black pop.	793	438	1.81 (1.69-1.94)	1.56	496,648	2,275,940
12.5%~<30.0%	106	58	1.82 (1.49-2.20)	1.42	79,498	336,694
30.0%~<50.0%	99	60	1.64 (1.33-2.00)	1.00	90,392	385,689
≥50.0%	19	5	3.51 (2.12-5.49)	3.27	10,005	41,551
Urban						
0%~<12.5% Black pop.	4,072	2,944	1.38 (1.34-1.43)	0.68	3,367,682	16,478,244
12.5%~<30.0%	778	679	1.14 (1.07-1.23)	0.25	828,102	3,985,623
30.0%~<50.0%	391	265	1.48 (1.33-1.63)	0.70	386,599	1,795,965
≥50.0%	97	58	1.67 (1.35-2.03)	0.82	102,734	473,722

SEER, Surveillance, Epidemiology, and End Results;
No., number; SMR, standardized mortality ratio; O/E, observed/expected; CI, confidence interval; EAR, excess absolute risk; pop., population
Rural, Non-metropolitan counties (Rural vs Urban Continuum codes 4-9); Urban, Metropolitan counties (Rural vs Urban Continuum codes 1-3)
Rural & 0-12.5% Black population: 258 counties; Rural & 12.5-30% Black population: 41 counties; Rural & 30-50% Black population: 52 counties; Rural & >50% Black population: 15 counties; Urban & 0-12.5% Black population: 155 counties; Urban & 12.5-30% Black population: 53 counties; Urban& 30-50% Black population: 27 counties; Urban & >50% Black population: 11 counties

© 2021 Suk R et al. *JAMA Network Open.*
